# Pathophysiological changes in incentive processing in episodic migraine: a preliminary event-related potential study

**DOI:** 10.1093/scan/nsaf039

**Published:** 2025-04-25

**Authors:** Cuihong Liu, Yue Qu, Guoliang Chen, Weiyan Ding, Edmund Derrington, Bing Zhang, Liyuan Pei, Yansong Li

**Affiliations:** Social Interaction and Learning Lab, Department of Psychology, School of Social and Behavioral Sciences, Nanjing University, Nanjing, 210023 China; Department of Psychiatry, The 967th Hospital of Joint Logistic Support Force of PLA, Dalian, 116011 China; Department of Psychiatry, The 967th Hospital of Joint Logistic Support Force of PLA, Dalian, 116011 China; Department of Psychiatry, The 967th Hospital of Joint Logistic Support Force of PLA, Dalian, 116011 China; Institute of Cognitive Science Marc Jeannerod, CNRS, Lyon, Université Claude Bernard Lyon 1, Villeurbanne, 69675 France; Department of Radiology, Nanjing Drum Tower Hospital, Affiliated Hospital of Medical School, Nanjing University, Nanjing, 210093 China; Institute for Brain Sciences, Nanjing University, Nanjing, 210023 China; Department of Psychiatry, The 967th Hospital of Joint Logistic Support Force of PLA, Dalian, 116011 China; Social Interaction and Learning Lab, Department of Psychology, School of Social and Behavioral Sciences, Nanjing University, Nanjing, 210023 China; Department of Radiology, Nanjing Drum Tower Hospital, Affiliated Hospital of Medical School, Nanjing University, Nanjing, 210093 China; Institute for Brain Sciences, Nanjing University, Nanjing, 210023 China

**Keywords:** migraine, reward, punishment, incentive, ERPs, MID

## Abstract

This study examined pathophysiological changes in incentive processing in migraineurs. Nineteen episodic migraine (EM) patients and 19 healthy controls (HCs) performed a monetary incentive delay task while their event-related potentials were recorded. During the incentive anticipation phase, both Cue-N2 and Cue-P3 amplitudes were responsive to incentive cues in both groups, indicating no between-group differences in the distinct anticipatory subprocesses that underly incentive cue evaluation. During the outcome phase, the feedback-related negativity amplitude, associated with performance evaluation, was larger for punishing feedback than rewarding feedback across both groups. However, the feedback-P3 amplitude, linked to attentional processing of motivational value of outcome feedback, was significantly larger for rewarding feedback than punishing feedback in HCs, but not in EM patients. Moreover, a significant negative correlation was observed between the feedback-P3 amplitude difference for rewarding minus punishing feedback and subjective pain intensity in EM patients. Finally, the feedback late-positive potential amplitude, related to affective processing of affective value of outcome feedback, was significantly larger for punishing feedback than rewarding feedback only in HCs, but not in EM patients. Our findings suggest that recurrent severe pain may relate to abnormal incentive-related brain activity during the outcome phase of incentive processing.

## Introduction

Migraine is a common neurological disease that typically causes moderate to severe headaches. It has been recognized to be an important medical issue that affects patients’ quality of life (Pietrobon and Moskowitz 2013, Arnold 2018). Over the past decades, increasing research efforts have been devoted to elucidating the pathogenetic mechanisms behind migraine by investigating the neural characteristics of migraine patients’ brains (Goadsby et al. 2009, May 2017, Sudershan et al. 2022). The majority of previous ERP studies have identified hypersensitivities to visual, auditory, somatosensory, and olfactory stimuli in the sensory domain in migraineurs, usually reflected by increased amplitude of evoked responses (Schwedt 2013, De Tommaso et al. 2014, Goadsby et al. 2017, Haigh et al. 2019).

Despite such advances, exploring how the migraine brain processes incentives (e.g. monetary rewards or punishment) has been largely ignored in previous research (Cahill et al. 2014). Such research might provide insight by identifying a possible role of dopamine (DA) in migraine pathophysiology (Mascia et al. 1998, Akerman and Goadsby 2007, Charbit et al. 2010, Cahill et al. 2014). The relevant literature shows that migraine is associated with the hypoactivation of dopaminergic neurons during the interictal period, which results in DA receptor hypersensitivity during headache attacks (Peroutka 1997, Fanciullacci et al. 2000, Chen 2006, Sokolov et al. 2018). Given that DA is an important neuromodulator that exerts widespread effects on both cortical and subcortical regions, where its receptors are widely distributed, a low DA tone may have profound effects on DA-mediated neurocircuitries in migraineurs. A few resting-state magnetic resonance imaging (MRI) studies provide limited but interesting evidence regarding dysregulation of DA-mediated neurocircuitry by showing aberrant functional connectivity within the meso-corticolimbic system in migraineurs in the interictal period relative to healthy controls (HCs) (Maleki et al. 2011, Kim et al. 2021a). Given the central role of this brain circuit in incentive processing (Lammel et al. 2014, Hu 2016), the altered functional connectivity within the meso-corticolimbic system raises the possibility that migraine may also be associated with pathological brain processing of incentives.

It is only in recent years that researchers have begun conducting empirical investigations to advance our understanding of the above issue. For example, a recent functional MRI (fMRI) study showed that, during the interictal period, episodic migraine (EM) patients have blunted neural responses to rewarding (monetary gains) and punishing (monetary losses) feedback during the outcome phase, but not during the anticipation phase (Kocsel et al. 2019). However, because fMRI lacks the temporal resolution to distinguish the rapidly unfolding stages within the anticipatory and consummatory phases of incentive processing, no work to date has examined potential migraine-related changes in neural processing during the successive stages of incentive anticipation and delivery.

The present study was designed to address this issue by using a combination of the monetary incentive delay (MID) task and event-related potentials (ERPs) in EM patients in the interictal period. In the MID task, participants were required to react to a target stimulus, presented after an incentive cue, to obtain a monetary incentive or to avoid losing the indicated incentive, a protocol similar to that described in previous ERP work (Flores et al. 2015). This task can allow the detailed examination of anticipatory and outcome-related ERP components during incentive processing in EM patients relative to HCs. Based on the current literature (Glazer et al. 2018), two commonly observed ERP components are particularly relevant during the anticipation phase: Cue-N2 and Cue-P3. The Cue-N2 refers to a negative-going wave that occurs 200–300 ms after cue onset, with its maximum amplitude over the frontocentral scalp region (Potts 2011, Santesso et al. 2012). The Cue-P3 is a positive-going wave that peaks from approximately 300 to 600 ms after the cue, with its maximum amplitude over the centro-parietal scalp region (Broyd et al. 2012, Pornpattananangkul and Nusslock 2015). Despite some ongoing debate concerning the functional significance of these two ERP components (Glazer et al. 2018), they are thought to reflect cue evaluation. Meanwhile, outcome-related ERPs can be separated into three different components that occur in different time windows: feedback-related negativity (FRN), Feedback P300 (FB-P3), and feedback late-positive potential (FB-LPP). The FRN is a negative-going wave that peaks around 200–300 ms after outcome feedback, over the frontocentral scalp region. Traditionally, the FRN is thought to be sensitive to performance evaluation during outcome processing (e.g. gain vs. loss or good vs. bad) (Sambrook and Goslin 2015). In addition, according to an influential theory (Holroyd and Coles 2002, Nieuwenhuis et al. 2004), the FRN codes a prediction error (PE) signal such that the FRN is sensitive to the unexpectedness of an outcome. The FB-P3, a centro-parietal positive-going deflection that occurs 300–600 ms after the outcome feedback, follows the FRN. This outcome-related ERP component is argued to involve attention-driven categorization of salient outcome-related information (Broyd et al. 2012, San Martín 2012). Finally, the FB-LPP, a centro-parietal positive-going wave, is the latest ERP during outcome processing, and occurs 500–800 ms after the FB-P3. Given its functional difference from the FB-P3 during outcome processing, it has been argued to reflect attentional processing of the affective value of outcome feedback (Groen et al. 2008, 2013). Analysis of these incentive-related ERPs may elucidate pathophysiological changes in incentive anticipation and its delivery in EM patients. Given that recent research has revealed blunted neural responses to rewarding and punishing stimuli, only during the outcome phase, but not during the anticipation phase in EM patients (Kocsel et al. 2019), we can expect dysfunctional outcome-related ERP components in EM patients compared to HCs.

## Materials and methods

### Participants

Our participant recruitment procedure was described at length in our recent study (Chen et al. 2020). Specifically, the patient group included 19 EM patients (mean age = 31.95 ± 1.42, 17 females). Each patient’s headache diary and structured questionnaires on demographics, headache profile, medical history, and medication use were recorded. The headache profile examined migraine history (years), the attack frequency (number per month) and duration of headaches (days per month), the presence of an aura (yes:no), the severity of migraine evaluated using a visual analogue scale (VAS), and included the six-item Headache Impact Test (HIT)-6, Self-rating Anxiety Scale (SAS), and Self-rating Depression Scale (SDS). The inclusion criteria for patients with EM were the following: (I) fulfilling the diagnostic criteria for migraine according to the International Classification of Headache Disorders, 3rd edition (ICHD-3) and (II) a history of at least 2 years of migraine and at least one migraine episode per month. Moreover, EM patients were excluded according to the following criteria: (I) neurological diseases (i.e. epilepsy, cerebral infarction, and encephalitis); (II) mental retardation; (III) a current or past history of substance dependence; (IV) receiving prophylactic anti-migraine therapy; and (V) depressive and anxiety disorders. Five migraineurs reported experiencing aura with their migraine, and the remaining 14 reported no aura. We also employed an age- and sex-matched HC group consisting of 19 healthy individuals (mean age = 30.16 ± 0.98, 16 females). None of them reported any personal or family history of psychiatric or neurological disorders, which was confirmed by both a self-reported past history and a psychiatric examination of the present mental state using the *Diagnostic and Statistical Manual of Mental Disorders*, fourth edition, (DSM-IV) criteria of axis I. None of the female participants from either group took any oral contraceptives for at least 1 week prior to involvement in this study. All participants in the two groups were right-handed. Informed consent was obtained from all participants prior to the experiment. The study protocol was approved by the Ethics Committee of the Chinese PLA General Hospital and conducted in accordance with the Declaration of Helsinki. Demographic and clinical characteristics are presented in Table 1.

**Table 1.** Demographic and clinical characteristics of the study sample.

	EM (*n* = 19; *M* ± SE)	HCs (*n* = 19; *M* ± SE)	Group comparison
Age, years	31.95 ± 1.42	30.16 ± 0.98	*t*(36) = 1.04, *P* > .05
Gender (F/M)	(17/2)	(16/3)	*P* > .05
Education, years	15.63 ± 0.71	14.53 ± 0.46	*t*(36) = 1.31, *P* > .05
BMI (kg/m^2^)	20.47 ± 0.70	20.44 ± 0.81	*t*(36) = 0.03, *P* > .05
Duration of migraine, days per month	13.37 ± 1.40		
History of migraine, years	12.74 ± 1.38		
Migraine frequency, times per month	4.39 ± 0.93		
Severity of headache (VAS scale)	7.84 ± 0.33		
HIT-6	63.84 ± 1.18		

VAS, with 0 indicating no pain and 10 as worst possible pain

### Monetary incentive delay task

**Figure 1.**
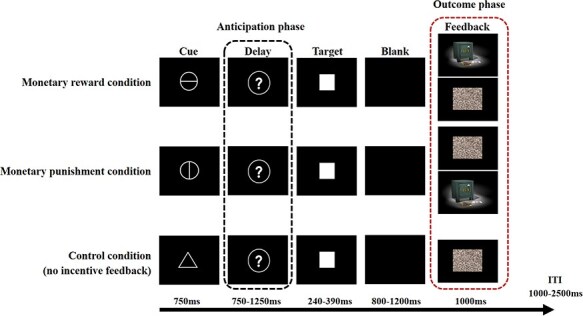
Trial sequence of the MID task. Each trial began with the presentation of a cue (a circle crossed by a horizontal line/a circle crossed by a vertical line/triangle) for 750 ms, to indicate the possible monetary reward/punishment/neutral feedback, respectively. After a variable delay period (750–1250 ms), during which a question mark was shown, a white square was presented as the target stimulus for 240–390 ms. The participants were required to respond by pressing a button as quickly as possible. Following an interval of 800–1200 ms, feedback was presented for 1000 ms. Each trial was followed by a blank screen for a variable intertrial interval (1000–2500 ms). The anticipation phase began after the presence of cue and the outcome phase began when feedback was presented .

We used a version of a MID task similar to that described in previous ERP studies (Flores et al. 2015). The MID task involved both monetary reward and punishment. In each trial, it included an anticipation phase, a target identification task, and an outcome phase (Fig. 1). During the anticipation phase, participants were presented one of three cues for 750 ms to inform them of the type of upcoming incentive. A circle crossed by a horizontal line indicated a potential monetary reward (+¥), and a circle crossed by a vertical line indicated a potential monetary punishment (−¥). In addition, a neutral cue (a triangle) was also included and signalled that there was no incentive outcome (reward/punishment). After a variable delay period (750–1250 ms), during which a question mark was shown, a white square was presented as the target stimulus for 240–390 ms. Similar to previous ERP work (Flores et al. 2015), the duration of this target stimulus was adjusted to produce a success rate of 55%– 65%. Participants were required to respond to the target by pressing a button as quickly as possible, which was followed by a delay of 800–1200 ms before the feedback was presented. If the participant responded to the target quickly enough after a reward-anticipation cue, a reward feedback (range: +¥10 to +¥20; mean: +¥15) displayed on a safe was presented. If they responded too slowly, then the no reward feedback (a ‘scrambled’ picture) was presented. Similarly, after a punishment-anticipation cue, if they responded to the target quickly enough (within the duration of the target onset) after a punishment-anticipation cue, no punishment feedback (‘scrambled’ picture) was displayed on a safe. However, if they did not respond fast enough, then the punishment feedback (range: −¥10 to −¥20; mean: −¥15) was presented. In trials with the neutral cue, participants were presented the ‘scrambled’ picture as feedback. Participants were told to avoid responding too quickly or too slowly. The feedback was presented for 1000 ms. Each trial was followed by a blank screen for a variable intertrial interval (1000–2500 ms). All responses within the duration of the target onset (240–390 ms) were designated as hits or accurate responses. Responses outside this time frame were considered inaccurate. Participants performed the task in three separate blocks of 100 trials each. Blocks were separated a short break. To familiarize participants with the task, the formal experiment was preceded by a short practice block. The total duration of the task was approximately 40 min.

### EEG data recording and analysis

Details of the electroencephalogram（EEG） data recording procedure were the same as that described in our recent studies (Chen et al. 2015, 2017). Specifically, we used a quick cap carrying 64 Ag/AgCl electrodes placed at standard locations covering the whole scalp (the extended international 10–20 system) to record EEG (Brain Products). The reference electrode was attached to the right mastoid (M2), and the ground electrode was placed on the forehead. The vertical electrooculogram (VEOG) was recorded with electrodes placed above and below the left eye. The horizontal electrooculogram (HEOG) was recorded with electrodes placed beside each eye. The impedance was kept below 5 kΩ. The electrophysiological data were recorded continuously with a bandwidth of 0.05–100 Hz and sampled at a rate of 1000 Hz.

Offline EEG data analysis was conducted using EEGLAB (Delorme and Makeig 2004) and ERPLAB (Lopez-Calderon and Luck 2014). Data were first re-referenced to linked mastoids (M1 and M2). An independent component analysis (ICA)-based artefact correction was achieved by using the ICA function of EEGLAB. Independent components with topographies representing saccades, blinks, and heart rate artefacts were thus removed according to published guidelines (Jung et al. 2000). The resultant EEG data were then epoched from 200 ms prestimulus to 800 ms poststimulus and digitally low pass filtered at 30 Hz (24 dB/octave). The 200 ms prestimulus period was used for baseline correction. In order to remove movement artefacts, epochs were rejected when fluctuations in potential values exceeded ±75 μV on any channel other than the EOG channel. In the anticipation phase, cue-locked ERPs were averaged separately for each anticipation cue type (reward vs. punishment vs. control) for each group (EM patients vs. HCs). In the feedback phase, feedback-locked ERPs were averaged for each outcome feedback type (rewarding feedback vs. punishing feedback vs. outcome feedback) in each group.

### Statistical analysis

With regard to statistical analysis of the demographic data, a chi-square test was employed to examine a between-group difference in sex ratio and independent sample t-tests were used to assess between-group differences in age, years of education, SAS scores, SDS scores, and body mass index (BMI). For statistical analysis of the behavioural data, both reactions times (RTs) and accuracy were analysed using a two-way mixed analysis of variance (ANOVA) with group (EM patients vs. HCs) as a between-participant factor and anticipation cue type (reward vs. punishment vs. control) as a within-participant factor.

For statistical analysis of electrophysiological data, our data were analysed according to the topographical distribution of grand averaged ERP activity as well as previous ERP studies (Santesso et al. 2012, Flores et al. 2015, Glazer et al. 2018). In the anticipation phase, the mean amplitude of the Cue-N2 was calculated by averaging values across the midline electrodes Fz, FCz, and Cz within the 250–400 ms time window after target stimulus onset, whereas the amplitude of the Cue-P3 was averaged across the midline electrodes Cz, CPz, and Pz within the 400–600 ms time window after target stimulus onset. To explore the effects of migraine on these incentive cue-related ERP components, we conducted two separate mixed ANOVA, with group as a between-participants factor (EM patients vs. HCs) and anticipation cue type (reward vs. punishment vs. neutral) as a within-participant factor. Moreover, in the feedback phase, like previous ERP work (Flores et al. 2015, Glazer et al. 2018), the mean amplitude of the FRN was calculated by averaging the values across the midline electrodes Fz, FCz, and Cz within the 210–310 ms time window after feedback delivery onset. In contrast, the mean amplitudes of the FB-P3 and FB-LPP were averaged across the midline electrodes Cz, CPz, and Pz within the 280–400 ms and 550–650 ms time windows after feedback delivery onset, respectively. To examine the effects of migraine on these outcome-related ERP components, we also conducted three mixed ANOVA, with group as a between-participants factor (EM patients vs. HCs) and outcome feedback type (rewarding feedback vs. punishing feedback vs. neutral feedback) as a within-participant factor. Finally, regarding ERP indices of incentive-anticipation cue and incentive feedback processing that show significant between-group differences, we further performed Pearson’s correlations to assess the possible relationships between these ERP indices and each of the seven clinical measures (SAS score, SDS score, frequency, duration, number of days, HIT-6, and VAS). To account for multiple correlations, the level of statistical significance for the correlation coefficients was adjusted by controlling the family-wise error rate (FWER) (Curtin and Schulz 1998).

All data were analysed using R. Statistical comparisons were made at *P*-values of *P* < .05, with the Greenhouse–Geisser correction when violations of sphericity occurred. Effect sizes for ANOVA effects (*η*2p) and t-tests (Cohen’s *d*) are provided.

## Results

### Behavioural results

The mixed ANOVA revealed a significant main effect of anticipation cue type on both RTs and accuracy (RTs: *F*(2, 72) = 7.74, *P* < .001, *η*2p = 0.18; Accuracy: *F*(2, 72) = 22.59, *P* < .001, *η*2 p = 0.39). *Post hoc* analysis revealed that RTs and accuracy on both reward (RTs: *t*(36) = −3.13, *P* = .005, *d* = −0.54; accuracy: *t*(36) = 5.45, *P* < .001, *d* = 1.02) and punishment trials (RTs: *t*(36) =  −3.13, *P* = .005, *d* = −0.58; Accuracy: *t*(36) = 4.92, *P* < .001, *d* = 0.89) were significantly faster and more accurate than those on neutral trials across both groups. However, our analysis did not reveal a significant main effect of group (RTs: *F*(1, 36) = 0.01, *P* = .99, *η*2 p = 0.000; accuracy: *F*(1, 36) = 0.02, *P* = .89, *η*2 p  = 0.001) or a significant group × incentive type interaction (RTs: *F*(2, 72) = 0.05, *P* = .96, *η*2 p  = 0.001; accuracy: *F* (2, 72) = 0.94, *P* = .38, *η*2 p  = 0.03).

### Electrophysiological results

#### ERP responses to incentive-anticipation cues

##### Cue-N2

Our mixed ANOVA revealed a significant main effect of anticipation cue type on the Cue-N2 amplitude (*F*(2, 72) = 8.06, *P* < .001, *η*2 p  = 0.18). Follow-up *post hoc* testing revealed that the Cue-N2 amplitude was highly sensitive to anticipation cue type, with decreased amplitudes for the punishment-anticipation cue compared to the reward-anticipation cue (*t*(36) = 2.78, *P* < .05, *d* = 0.37), and the neutral cue (*t*(36) = 3.69, *P* < .005, *d* = 0.66). However, there was no significant main effect of group (*F*(1, 36) = 1.17, *P* = .29, *η*2 p  = 0.03), nor a significant interaction between group and anticipation cue type (*F*(2, 72) = 0.46, *P* = .63, *η*2 p  = 0.01).

##### Cue-P3

Regarding the Cue-P3, our mixed ANOVA revealed a significant main effect of anticipation cue type on the Cue-P3 amplitude (*F*(2, 72) = 3.01, *P* = .05, *η*2 p  = 0.08). The *post hoc* testing revealed that the Cue-P3 amplitude was also sensitive to anticipation cue type, with its amplitude being larger for the punishment-anticipation cue than for the neutral cue (*t*(36) = 2.90, *P* < .05, *d* = 0.40). However, there was no significant main effect of group (*F*(1, 36) = 0.01, *P* = .94, *η*2 p  = 0.001), nor a significant interaction between group and anticipation cue type (*F*(2, 72) = 1.80, *P* = .18, *η*2 p  = 0.05).

#### ERP responses to incentive feedback

##### FRN

**Figure 2.**
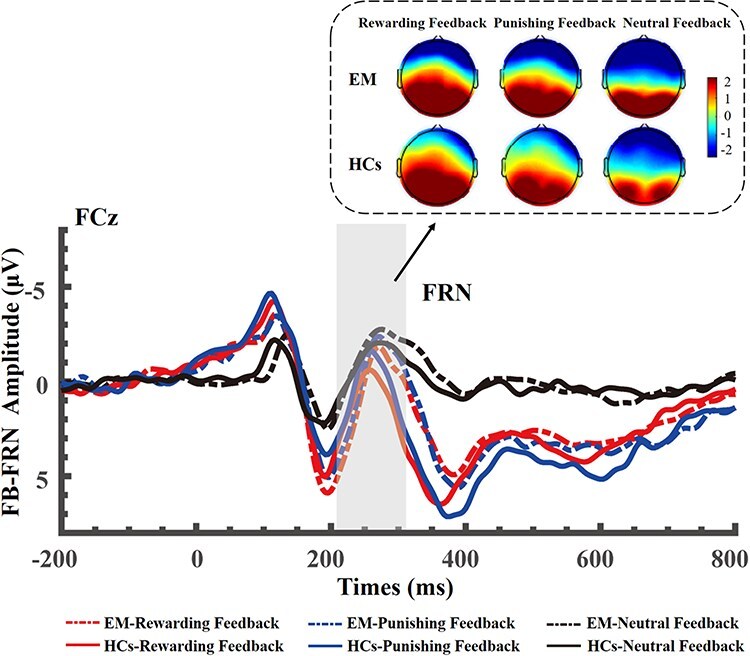
The FRN related to performance evaluation. Grand-average ERP waveforms recorded at FCz elicited by rewarding feedback, punishing feedback and neutral feedback, and topographical distribution of the FRN (210–310 ms) in EM patients and HCs. Positive isopotential lines are in red and negative isopotential lines are in blue.

Concerning the FRN, our mixed ANOVA showed a significant main effect of outcome feedback type on its amplitude (*F*(2, 72) = 15.64, *P* < .001, *η*2 p  = 0.30) (Fig. 2). The *post hoc* testing revealed that both rewarding (*t*(36) = 4.46, *P* < .001, *d* = 0.90) and punishing feedback (*t*(36) = 3.59, *P* = .001, *d* = 0.62) elicited a significantly larger FRN amplitude than the neutral feedback. Moreover, punishing feedback evoked a significantly larger FRN amplitude than the rewarding feedback (*t*(36) = −2.70, *P = *.01, *d* = −0.29). However, there was no significant main effect of group (*F*(1, 36) = 0.24, *P* = .63, *η*2 p  = 0.01), nor a significant interaction between group and outcome feedback type (*F*(2, 72) = 0.01, *P *= .96, *η*2 p  = 0.001).

##### FB-P3

**Figure 3.**
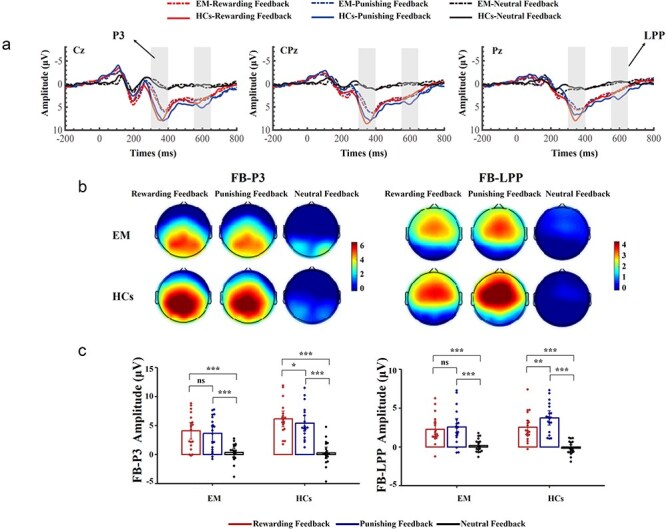
The feedback-P3 and feedback-LPP related to processing of motivational and affective value of outcome feedback. (a) The feedback-P3 (280–400 ms) and feedback-LPP (550–650 ms) over the central, parieto-central, and parietal regions (Cz, CPz, and Pz) for rewarding feedback, punishing feedback, and neutral feedback trials in EM patients and HCs. (b) Topographical maps of the voltage amplitudes for rewarding feedback, punishing feedback, and trials in the two groups for feedback-P3 (left) and feedback-LPP (right). Negative isopotential lines are in blue and positive isopotential lines are in red. (c) Means of the amplitudes of the feedback-P3 (left) and feedback-LPP (right) in the two groups. Error bars indicate 95% confidence interval; *P < .05, **P < .01, ***P < .001.

Regarding FB-P3, our mixed ANOVA showed a significant main effect of outcome feedback type on its amplitude (*F*(2, 72) = 113.79, *P* < .001, *η*2 p  = 0.76), whereas the main effect of group was not significant (*F*(1, 36) = 2.96, *P* = .09, *η*2 p  = 0.08). The *post hoc* testing revealed that both rewarding (*t*(36) = 11.84, *P < *.001, *d* = 2.28) and punishing feedback (*t*(36) = 10.86, *P < *.001, *d *= 1.99) elicited a significantly larger FB-P3 amplitude than neutral feedback. Moreover, rewarding feedback evoked a significantly larger FB-P3 amplitude than punishing feedback (*t*(36) = 2.78, *P < *.01, *d* = 0.29). This effect is further characterized by a significant interaction between group and outcome feedback type (*F*(2, 72) = 5.63, *P* < .01, *η*2 p  = 0.14) (Fig. 3). Simple effects analysis revealed a significant effect of outcome feedback type at each level of the group factor (EMs: *F*(2, 36) = 21.48, *P < *.001, *η*2 p  = 0.54; HCs: *F*(2, 36) = 53.21, *P < *.001, *η*2 p  = 0.75). Furthermore, while both rewarding (EMs: *t*(36) = 6.50, *P < *.001, *d* = 1.77; HCs: *t*(36) = 10.24, *P < *.001, *d* = 2.79) and punishing feedback (EM patients: *t*(36) = 5.99, *P < *.001, *d* = 1.55; HCs: *t*(36) = 9.37, *P < *.001, *d* = 2.43) elicited significantly larger FB-P3 amplitudes than neutral feedback in both groups, rewarding feedback evoked a significantly larger FB-P3 amplitude than punishing feedback in HCs (*t*(36) = 2.45, *P <* .05, *d* = 0.36), but not in EM patients (*t*(36) = 1.47, *P = *.15, *d* = 0.21) (Fig. 3).

##### FB-LPP

With regard to the FB-LPP, a significant main effect of outcome feedback type on its amplitude was observed (*F*(2, 72) = 58.41, *P* < .001, *η*2 p  = 0.62), whereas the main effect of group was not significant (*F*(1, 36) = 0.79, *P* = .38, *η*2 p  = 0.02). Follow-up *post hoc* testing revealed that both rewarding (*t*(36) = 8.29, *P <* .001, *d* = 1.30) and punishing feedback (*t*(36) = 9.40, *P <* .001, *d* = 1.70) elicited significantly larger FB-LPP amplitudes than neutral feedback. Additionally, punishing feedback evoked a significantly larger FB-LPP amplitude than rewarding feedback (*t*(36) = 2.63, *P <* .05, *d* = 0.41). Moreover, a significant interaction between group and outcome feedback type was observed (*F*(2, 72) = 3.15, *P* < .05, *η*2 p  = 0.09) (Fig. 3). Simple effects analysis revealed a significant effect of outcome feedback type at each level of the group factor (EMs: *F*(2, 36) = 16.20, *P < *.001, *η*2 p = 0.47; HCs: *F*(2, 36) = 36.36, *P < *.001, *η*2 p = 0.67). Moreover, while both rewarding (EM patients: *t*(36) = 5.10, *P <* .001, *d* = 1.13; HCs: *t*(36) = 6.61, *P <* .001, *d* =1.46) and punishing feedback (EM patients: *t*(36) = 5.04, *P <* .001, *d* = 1.29; HCs: *t*(36) = 8.25, *P <* .001, *d* = 2.11) evoked significantly larger FB-LPP amplitudes than neutral feedback across both groups, punishing feedback evoked a significantly larger FB-LPP amplitude than rewarding feedback only in HCs (*t*(36) = 2.97, *P <* .01, *d* = 0.65), but not in EM patients (*t*(36) = 0.75, *P = *.46, *d* = 0.16) (Fig. 3).

#### Brain–behaviour relationships

**Figure 4.**
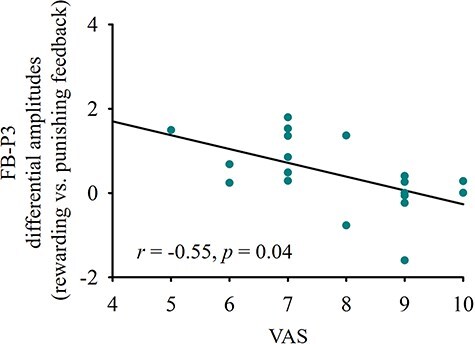
Brain–behaviour correlation. A significant negative correlation was found between the difference scores of FB-P3 amplitudes to rewarding versus punishing feedback, computed as electrode averages across Cz and CPz, and VAS in EM patients.

To examine brain–behaviour relationships in EM patients, we correlated FB-P3 mean values in response to rewarding and punishing feedback separately, as well as the difference scores of FB-P3 amplitudes (rewarding vs. punishing feedback) with each clinical measure (SAS score, SDS score, frequency, duration, number of days, HIT-6, and VAS). Difference scores were computed regarding the FB-P3 and FB-LPP amplitudes because our ERP analyses revealed within-group differences between the rewarding and punishing feedback in EM patients. For each of the seven clinical measures, three pairwise correlations were conducted, collectively forming a family of tests. Consequently, the multiple comparison correction based on FWER was applied within each set of three pairwise correlations. Similarly, the same analyses were conducted for FB-LPP. These analyses revealed a significant negative correlation between the difference scores of FB-P3 amplitudes to rewarding versus punishing feedback and VAS (*r* = −0.55, *P* = .04) (Fig. 4).

## Discussion

To our knowledge, this preliminary ERP study is the first to investigate incentive-related neural activity along the temporal scale in EM patients in the interictal period and HCs. At the neural level, in the anticipation phase, both Cue-N2 and Cue-P3 amplitudes were larger for the reward-anticipation and punishment-anticipation cues compared to the neutral cues for both groups. This result confirmed previous findings showing that during the interictal period, EM patients do not show blunted neural responses, as measured by fMRI, in anticipation of either reward or punishment (Kocsel et al. 2019). However our study did reveal dysfunctional outcome-related ERP components in EM patients. Given these findings, we will focus on these outcome-related ERP components along the temporal scale of outcome evaluation and discuss possible implications for advancing our understanding of migraine pathophysiology.

The first substage of outcome evaluation can be quantified through the FRN (Miltner et al. 1997). Most previous ERP studies have demonstrated that the FRN tends to be smaller for positive performance feedback (i.e. gains) than for negative performance feedback (i.e. losses) (Glazer et al. 2018). This reflects a binary evaluation of positive versus negative performance feedback (Hajcak et al. 2006, San Martín 2012, Walsh and Anderson 2012). In this sense, the FRN is often argued to be an indicator of positive outcome feedback during outcome evaluation and, consequently, to be particularly sensitive to performance evaluation (Glazer et al. 2018). Our results regarding the FRN suggest that EM patients retained the ability to distinguish between outcome feedback indicating good and bad performance. This is reflected by the significant main effect of outcome feedback type across both groups, and the absence of a significant interaction between outcome feedback type and group. In addition, according to an influential theory, the FRN, after the delivery of a probabilistic incentive, codes a PE signal such that the FRN is larger for outcomes that are worse than expected (Holroyd and Coles 2002, Nieuwenhuis et al. 2004). Our observation that the FRN is larger for punishing feedback than for rewarding feedback across both groups is consistent with this theory. Meanwhile, according to source localization, the FRN is thought to index activity in the medial prefrontal cortex (mPFC), most probably in the anterior cingulate cortex (ACC) (Gehring and Willoughby 2002). In this case, it may be speculated that EM patients, compared to HCs, do not show a notable functional impairment in processing negative or positive outcomes (i.e. losses or gains) within the ACC. Indeed, the major findings of a recent fMRI study seems to support this assumption by showing that EM patients did not display alterations in the ACC activity related to processing gains and losses compared to HCs (Kocsel et al. 2019). Our preliminary results concerning the FRN suggest that neurophysiological responses to the valence of outcome feedback during the early stage of outcome evaluation appear to be preserved in EM patients.

The ERP component typically used to quantify the second substage of outcome evaluation is the FB-P3. The FB-P3 is often argued to be sensitive to the motivationally salient nature of outcome feedback (San Martín 2012, Glazer et al. 2018). Hence, the FB-P3 should be primarily modulated by reward evaluation such that incentive feedback (reward and punishment) would enhance the FB-P3 relative to the control/neutral feedback (Van den Berg et al. 2012). Our results concerning the FB-P3 showed that both rewarding feedback and punishing feedback were associated with enhanced FB-P3 positivity relative to the neutral feedback across both groups. Thus, EM patients were able to discriminate between incentive feedback (reward and punishment) and neutral feedback in terms of motivational salience. Furthermore, the motivational salience of incentive feedback seems to scale with incentive valence. Despite some controversy (Campbell et al. 1979, Yeung and Sanfey 2004, Gu et al. 2011, San Martín 2012), most ERP studies have consistently reported that positive feedback evokes a more positive FB-P3 than negative feedback (Hajcak et al. 2007, Bellebaum et al. 2010, Polezzi et al. 2010, Kreussel et al. 2012). Given that there is overwhelming evidence supporting a motivational salience account of attentional control (Donchin and Coles 1988, Nieuwenhuis et al. 2005, Kim et al. 2021b), this effect has been interpreted to reflect attention-driven categorization of motivationally salient outcome-related information (San Martín 2012). According to this interpretation, the enhanced positivity for rewarding feedback compared to punishing feedback reflects increased attention to the motivational salience of rewarding feedback relative to punishing feedback. In the present study, the influence of incentive valence on the amplitude of the FB-P3 in HCs was consistent with previous findings. However, the FB-P3 amplitude for rewarding and punishing feedback did not differ significantly in EM patients. This blunted difference in FB-P3 amplitude may indicate a potential difficulty in distinguishing the motivational salience of rewarding and punishing feedback during incentive evaluation in EM patients. Moreover, this blunting appeared to be linked to the severity of migraine-related symptoms, as indicated by a significant negative correlation between the FB-P3 amplitude difference (rewarding minus punishing feedback in the same EM patient) and the subjective pain intensity of their migraine during attacks, as measured by the VAS in these patients. Thus, the larger the blunting of the difference in FB-P3 amplitude between rewarding and punishing feedback, the higher the subjective evaluation of pain intensity by EM patients. In addition, according to source localization, the FB-P3 is commonly thought to index activity in multiple neural sources but particularly the temporoparietal junction (TPJ), anterior insula (AI), and amygdala. Most of these brain areas are key components of the salience network (SN), which plays a crucial role in assigning motivational salience to incoming stimuli (Bressler and Menon 2010). Therefore, our preliminary results may offer additional EEG-based insights into how migraine could influence neural activities in the SN. Within this domain, our recent resting-state EEG study has found a reduction in neural activities in the SN at baseline in migraineurs as captured by the significantly decreased presence of microstate class C (Li et al. 2022). This indicates a potential alteration in the processing of motivationally salient information in migraineurs. By taking advantage of task-oriented EEG, our present preliminary study has provided direct neural evidence supporting this argument by showing relatively blunted neurophysiological responses to rewarding versus punishing feedback in EM patients.

Following the FB-P3, the final ERP component during outcome evaluation is the FB-LPP, which is a positive-going centroparietal ERP component that follows outcome presentation (Schupp et al. 2006). Due to the fact that the FB-LPP is not typically studied in the context of incentive processing, there seems to be a very limited understanding of its functional significance and its functional differences from the FB-P3 during outcome evaluation (Glazer et al. 2018). Despite this, given that the FB-LPP displays a similar scalp topography and functionality as the relatively well-studied FB-P3, it is commonly believed that the FB-LPP is an affective counterpart of the FB-P3 (Groen et al. 2008, 2013). Thus, the FB-LPP has been interpreted to reflect extended cognitive and attentional processing of the affective value of the outcome feedback (Glazer et al. 2018). As a consequence, like the FB-P3, the FB-LPP has also been found to be sensitive to reward evaluation (Pornpattananangkul and Nusslock 2015). Our preliminary findings regarding the FB-LPP suggest that both rewarding and punishing feedback elicited significantly larger FB-LPP amplitudes compared to neutral feedback across both groups. These results indicate that EM patients retained the ability to distinguish between incentive feedback (reward and punishment) and neutral feedback in terms of affective value. Moreover, despite limited evidence, a few ERP studies have further found the influence of incentive valence on the FB-LPP by showing enhanced positivity following negative feedback over positive feedback (van Meel et al. 2005, Groen et al. 2008, Pornpattananangkul and Nusslock 2015, Donaldson et al. 2016). In the present study, such a negativity bias was observed in HCs but was absent in EM patients. This preliminary finding suggests a potential impairment in distinguishing the affective value of rewarding versus punishing feedback during outcome evaluation in these patients.

### Potential limitation

In spite of our promising findings, there were several potential limitations of this study that need to be indicated. First, the relatively small sample size in this study limits the statistical power to detect complex interactions. Therefore, our findings should be interpreted with caution. Nonetheless, this study provides valuable insights and lays the foundation for future research, which should include larger sample sizes to validate these results. Second, EM can be divided into low frequency and high frequency according to headache frequency. Our present study only included patients with low-frequency EM (headache frequency was around 4 times per month). Considering that high and low-frequency EM have been revealed to differ regarding the burden of headache, quality of life, psychological burden, and presence of symptoms related to sensitization (Di Antonio et al. 2021), this may affect the “generalizability” of our findings in migraineurs. Future research should explore the similarities and differences in pathophysiological signatures of incentive processing across EM phenotypes. Third, the majority of EM patients (17/19), in the present study were female. Due to the fact that differences between male and female migraine patients have been identified at both behavioural and brain levels (Maleki et al. 2012, Maleki and Androulakis 2019, Al-Hassany et al. 2020), it remains unclear whether these findings can be generalized to male migraine patients. Future research should take this into account. Fourth, in our correlation analyses, the multiple comparison correction based on FWER was applied only within each set of three pairwise correlations. This adjustment may not have been sufficiently stringent, potentially affecting the robustness of the correlation results. Therefore, our correlational findings require further validation in future studies.

## Conclusion

The present study was designed to characterize pathophysiological features of incentive processing using ERPs in the interictal period in EM patients. Migraine-related changes in ERP components implicated in outcome evaluation were observed. Specifically, although rewarding and punishing feedback elicited larger FB-P3 and FB-LPP amplitudes than the neutral feedback across both groups, the difference in FB-P3 and FB-LPP amplitudes between rewarding and punishing feedback was found to be blunted in EM patients. These findings suggest a potential difficulty in differentiating the motivational salience and the affective value of rewarding versus punishing feedback during incentive evaluation in these patients. The present study offers novel insights into the temporal dynamics of migraine-related changes in incentive processing in migraineurs. In addition, our findings may provide valuable insights for future research aimed at understanding altered incentive processing in migraineurs better and evaluating how clinical interventions help restore normal outcome processing in EM patients.

## Data Availability

The datasets used and/or analysed during the current study could be available from the corresponding author on a reasonable request.
